# Supplementary material for: COVID-19 Is a Confounder of Increased Candida Airway Colonisation
Source: Pathogens. 2023 Mar 15;12(3):463. doi: 10.3390/pathogens12030463 (PMC10052038; doi:10.3390/pathogens12030463)
Supplement: Supplementary file 1 [file pathogens-12-00463-s001.zip › pathogens-2198696-supplementary/Table S1.docx]

**Table S1**. Antibacterials used in the case and control groups: Tbact (patients with positive bacterial culture) and Tneg (patients with negative respiratory sample cultures).

| **Antibacterial therapy, n (%)** | **Cases (n=100)** | **Tbact (n=100)** | **Tneg (n=100)** |
| --- | --- | --- | --- |
| Group A penicillins | 12 (12) | 23 (23) | 13 (13) |
| Oxacillin IV^∇^ | 8 (8) | 2 (2) | 1 (1) |
| Cefotaxime, Ceftriaxone | 18 (18) | 23 (23) | 11 (11) |
| Ceftazidime | 23 (23) | 20 (20) | 0 (0) |
| Cefepime | 30 (30) | 17 (17) | 2 (2) |
| Linezolid | 28 (28) | 13 (13) | 6 (6) |
| Macrolides | 2 (2) | 5 (5) | 7 (7) |
| Clindamycin | 6 (6) | 2 (2) | 1 (1) |
| Fluoroquinolones | 10 (10) | 13 (13) | 5 (5) |
| Bactrim | 5 (5) | 6 (6) | 2 (2) |
| Metronidazole | 5 (5) | 7 (7) | 1 (1) |
| Piperacillin/tazobactam | 44 (44) | 34 (34) | 14 (14) |
| Carbapenems | 35 (35) | 21 (21) | 2 (2) |
| Vancomycin | 13 (13) | 10 (10) | 3 (3) |
| Daptomycin | 4 (4) | 3 (3) | 0 (0) |
| Aminoglycosides | 31 (31) | 23 (23) | 4 (4) |
| Others^‡^ | 7 (7) | 5 (5) | 0 (0) |

∇: Amoxicillin and Amoxicillin+Clavulanic acid;

IV: intravenous;

‡: Cefiderocol, Ceftazidime/Avibactam, Rifampin, Temocillin, Tigecycline.
