# Supplementary figures and images for: COVID-19 Is a Confounder of Increased Candida Airway Colonisation
Source: Pathogens. 2023 Mar 15;12(3):463. doi: 10.3390/pathogens12030463 (PMC10052038; doi:10.3390/pathogens12030463)

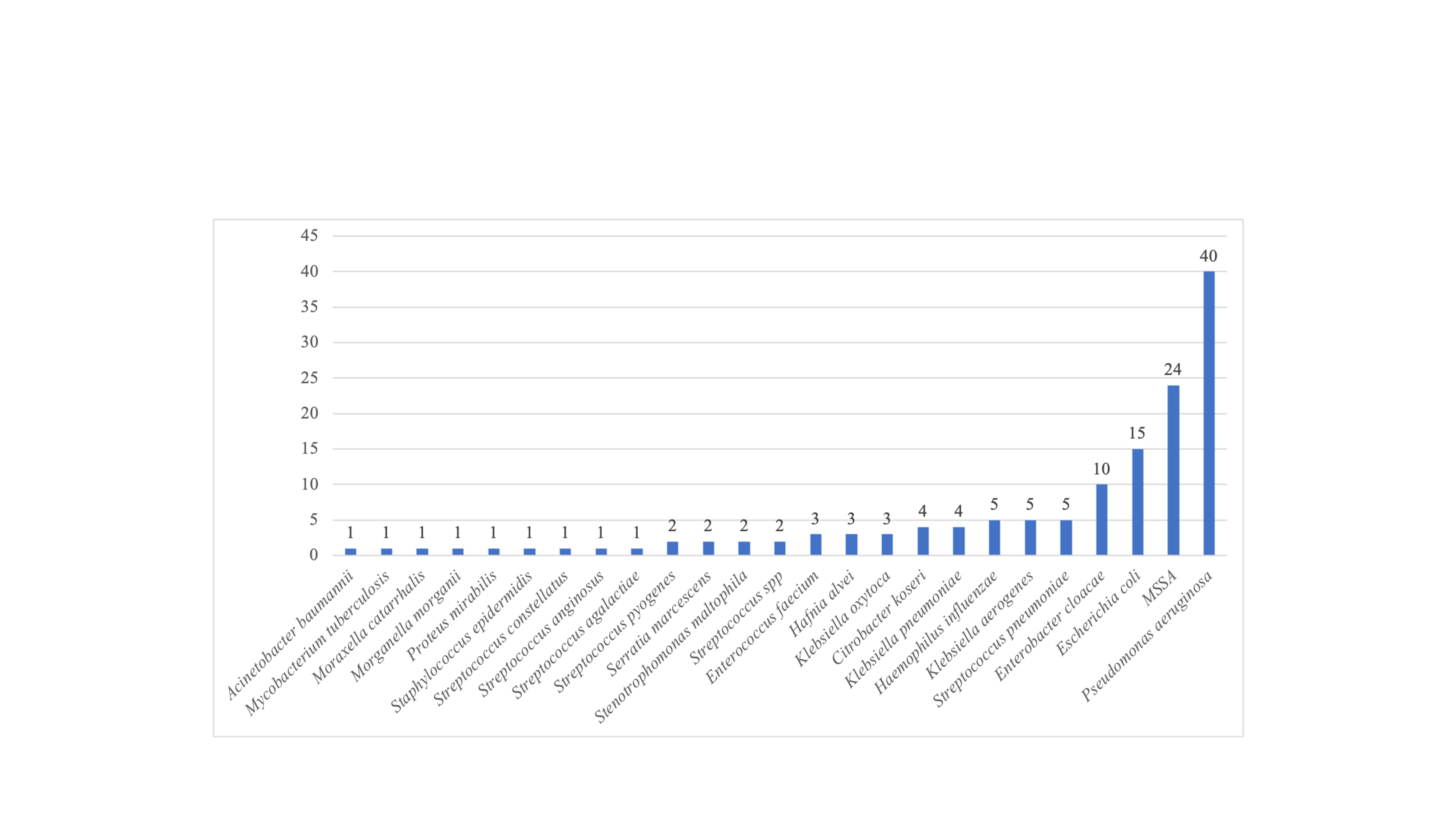

Supplement: Supplementary file 1 [file pathogens-12-00463-s001.zip › Fig S1.TIF]

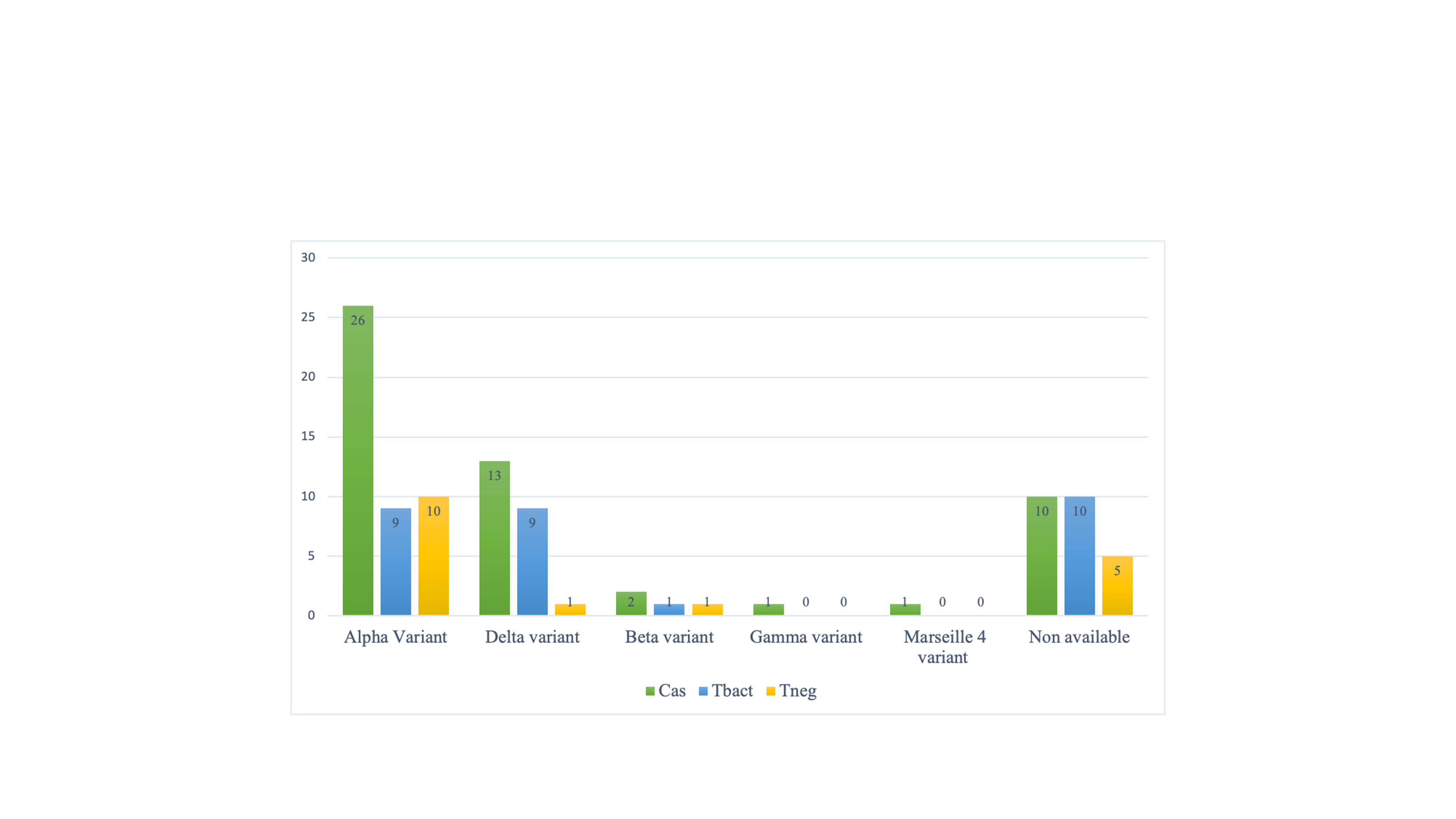

Supplement: Supplementary file 1 [file pathogens-12-00463-s001.zip › Fig S2.TIF]

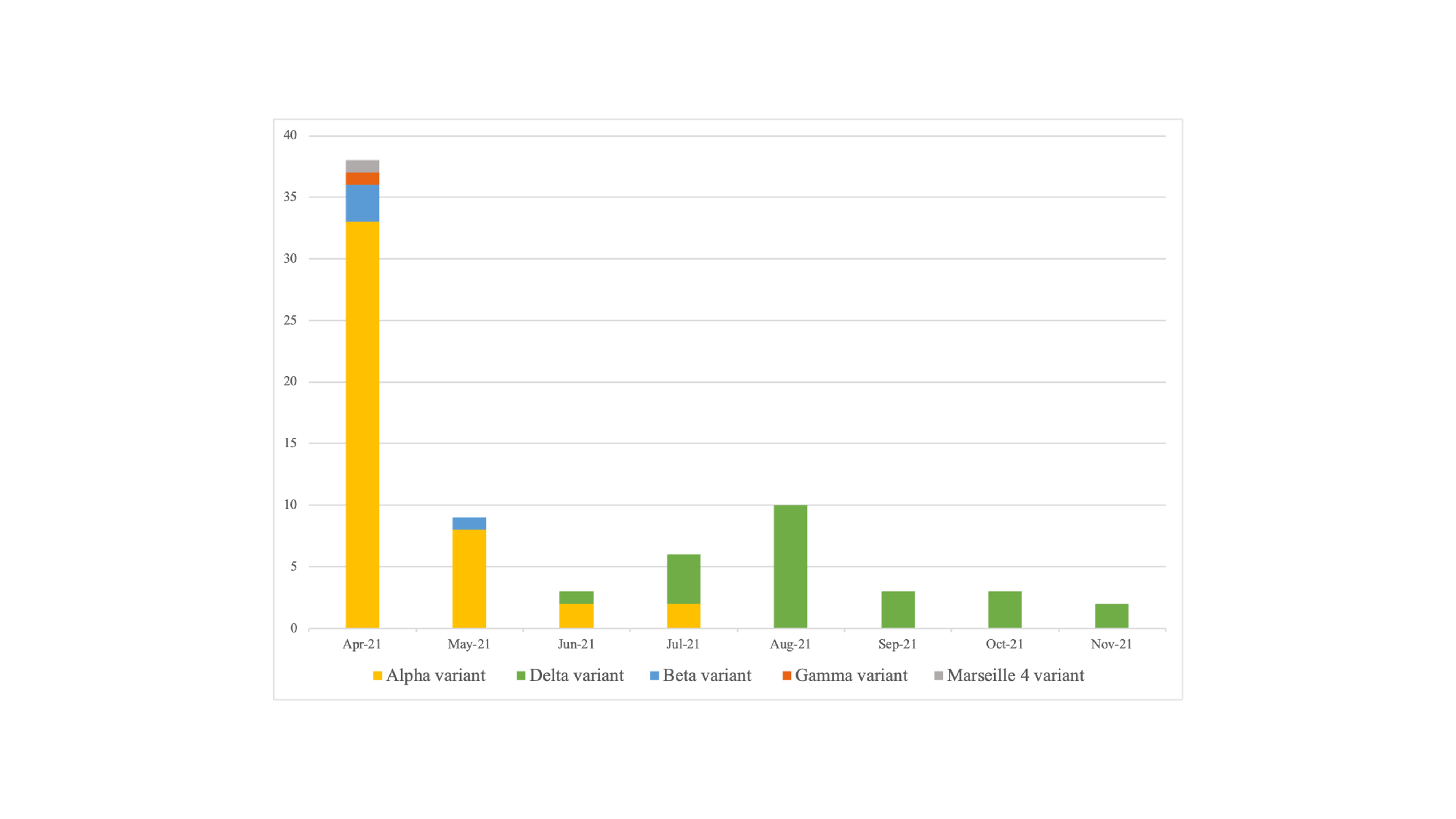

Supplement: Supplementary file 1 [file pathogens-12-00463-s001.zip › Fig S3.TIF]
